# Ultra-low-dose computed tomography and chest X-ray in follow-up of high-grade soft tissue sarcoma—a prospective comparative study

**DOI:** 10.1038/s41598-024-57770-z

**Published:** 2024-03-26

**Authors:** Samuli Salminen, Sari Jäämaa, Riikka Nevala, Markus J. Sormaala, Mika Koivikko, Erkki Tukiainen, Jussi Repo, Carl Blomqvist, Mika Sampo

**Affiliations:** 1 Comprehensive Cancer Center, Helsinki University Hospital (HUH), Helsinki, Finland; 2 University of Helsinki, Helsinki, Finland; 3 Department of Radiology, Helsinki University Hospital, Meilahti Campus Topeliuksenkatu 32, N0029 Helsinki, Finland; 4 Department of Plastic Surgery, Helsinki University Hospital and University of Helsinki, Helsinki, Finland; 5 Department of Orthopedics and Traumatology, Tampere University Hospital and University of Tampere, Tampere, Finland; 6 HUSLAB Pathology and University of Helsinki, Helsinki, Finland

**Keywords:** Cancer imaging, Sarcoma

## Abstract

Ultra-low-dose computed tomography (ULD-CT) may combine the high sensitivity of conventional computed tomography (CT) in detecting sarcoma pulmonary metastasis, with a radiation dose in the same magnitude as chest X-ray (CXR). Fifty patients with non-metastatic high-grade soft tissue sarcoma treated with curative intention were recruited. Their follow-up involved both CXR and ULD-CT to evaluate their different sensitivity. Suspected findings were confirmed by conventional CT if necessary. Patients with isolated pulmonary metastases were treated with surgery or stereotactic body radiation therapy (SBRT) with curative intent if possible. The median effective dose from a single ULD-CT study was 0.27 mSv (range 0.12 to 0.89 mSv). Nine patients were diagnosed with asymptomatic lung metastases during the follow-up. Only three of them were visible in CXR and all nine in ULD-CT. CXR had therefore only a 33% sensitivity compared to ULD-CT. Four patients were operated, and one had SBRT to all pulmonary lesions. Eight of them, however, died of the disease. Two patients developed symptomatic metastatic recurrence involving extrapulmonary sites+/−the lungs between two imaging rounds. ULD-CT has higher sensitivity for the detection of sarcoma pulmonary metastasis than CXR, with a radiation dose considerably lower than conventional CT.

Clinical trial registration: *NCT05813808. 04-14-2023*.

## Introduction

Approximately one half of patients with high-grade soft tissue sarcoma develop distant metastases, most commonly in the lungs^1^. If pulmonary recurrence is detected at a stage where it can be resected with clean margins, a proportion of patients may be cured of their disease. Many studies have shown that a considerable proportion of sarcoma patients operated for lung metastases are long-term survivors^2^. In a study from our own institution, 17% of patients with complete resection of lung metastases from soft tissue sarcoma remained free from further relapse for 5 to 10 years after resection and were thus probably cured of their disease^3^. Recently, stereotactic body radiation therapy (SBRT) has emerged as another possibility of achieving long-term tumour control in patients with pulmonary metastases from sarcoma^4^. If surgery or SBRT is not possible metastatic sarcoma is incurable. The rationale for intensive screening for sarcoma pulmonary metastasis is to enable treatment with curative intent even in the case of metastatic disease.

High-level evidence for the implementation of follow-up and its benefit in soft tissue sarcoma is scarce^5^. In a randomized 500-patient study, pulmonary relapse was detected earlier on chest CT than on plain X-ray (CXR), but this did not translate into a statistically significant overall survival advantage^6,7^. Lack of high-level evidence is also evident in the common National Comprehensive Cancer Network guidelines (NCCN), which states that CT is the preferred imaging modality, but “plain X-rays may be substituted for long-term survivors on surveillance to minimize radiation exposure from CT imaging^8^”. The European Society of Medical Oncology (ESMO), European Reference Network on Rare Adult Cancers (EURACAN), and European Reference Network on GENetic TUmour RIsk Syndromes (GENTURIS) guidelines only state that the use of CT for pulmonary metastases is likely to find recurrences earlier than other assessment/imaging modalities^9^.

As the growth rate of pulmonary metastases from sarcomas can be rapid, with minimum doubling times of only 7 to 9 days^10,11^ and the detection limit of roughly 10 mm of CXR, a metastasis may grow to considerable size between two controls. As the detection limit of CT is considerably lower (2 mm to 4 mm), the use of CT and shorter interval in follow-up might prevent the development of inoperable disease between two subsequent controls, and thus enable surgery in more patients.

There is growing evidence from epidemiological data that CT scans can cause cancer. A review of 17 epidemiological studies on assessing cancer risks from CT scans using medical record linkage showed wide variability in methodology across studies^12^. Organ-specific dose–response estimates, when reported, were, however, statistically compatible across studies and the summary excessive relative risk (ERR) per 100 mGy was significantly increased for both childhood leukemia (ERR/100 mGy was 1.78 (95%CI 0.01–3.53)) and brain tumors (0.80 (95%CI 0.48–1.12)). Conclusion of the meta-analyses was that increased CT imaging poses an increased risk for cancer, but the absolute risks are likely to be small, and repeated CTs are justifiable when clinically indicated, and the dose optimized.

Imaging based on traditional back-projection computation requires a larger beam dose than CXR. The development of CT imaging has brought new possibilities. The new model-based iterative image reconstruction (MBIR) calculation can be used to produce CT images of the lungs at significantly lower beam doses^13^. MBIR enables ultra-low-dose diagnostic computed tomography (ULD-CT) of the chest scans with an effective beam dose of about 0.3–0.4 mSv, which approaches the dose of CXR (0.1 mSv). Current advances in artificial intelligence will probably yield ULD-CTs with even further dose reductions^14^ and either augment or automate the detection and interpretation of small pulmonary metastases^15,16^. In comparison, conventional diagnostic pulmonary CT scan causes an effective beam dose of about 3 mSv^17^. ULD-CT has previously been found to be a sensitive method for detecting lung nodules^18^.

The aims of this prospective comparative study were to investigate whether ULD-CT is more accurate than CXR in the follow-up of soft tissue sarcoma and especially, whether the most fast-growing pulmonary metastases could be detected earlier. To enable optimal comparison of the two imaging methods, ULD-CT and CXR were performed to the patients during the same visit to the radiology department. Imaging was done according to a pre-defined schedule, with the most frequent imaging studies in the beginning of follow-up.

## Methods

### Treatment and follow-up protocol for soft tissue sarcoma in Helsinki University Hospital

Our treatment protocol for soft tissue sarcoma was established in 1987. The individual treatment plan for each new soft tissue sarcoma patient is decided at weekly meetings of a multidisciplinary team. Staging procedures include magnetic resonance imaging (MRI) or CT or both of the primary tumour and an ultrasound-guided or CT-guided core needle biopsy. A contrast-enhanced conventional CT of the chest is performed to all patients for staging purposes. Patients undergo contrast-enhanced conventional CT or MRI of other sites only if clinically indicated. Surgery with wide margins is preferred when feasible. Tumours are classified according to the latest WHO classification of tumours^19^. Radiotherapy is recommended after marginal surgery. High-risk patients with WHO performance status 0–1^20^ are also offered adjuvant chemotherapy consisting of six cycles of doxorubicin and ifosfamide with 21-day intervals.

After primary staging and treatment, patients remain on scheduled follow-up. Patients with high-grade sarcoma undergo a CXR every two months during the first two years, and thereafter three times annually up to five years (Table 1). Physical examination and a CT or an MRI scan of the primary tumour region are done six months postoperatively and thereafter once every six months until the two-year control and thereafter annually until the five-year visit.

**Table 1:** Current routine follow-up program for patients with high-grade soft tissue sarcoma and study program.

Timing	Routine follow-up program	Study follow-up program
2 months	CXR	CXR, ULD-CT
4 moths	CXR	CXR, ULD-CT
6 months	CXR, MRI or CT of the primary site, PE	CXR, ULD-CT, MRI or CT of the primary site, PE
8 months	CXR	CXR
10 months	CXR	CXR, ULD-CT
12 months	CXR, MRI or CT of the primary site, PE	CXR, MRI or CT of the primary site, PE
14 months	CXR	CXR, ULD-CT
16 months	CXR	CXR
18 months	CXR, MRI or CT of the primary site, PE	CXR, ULD-CT, MRI or CT of the primary site, PE
20 months	CXR	CXR
22 months	CXR	CXR
2 years	CXR, MRI or CT of the primary site, PE	CXR, ULD-CT, MRI or CT of the primary site, PE
	↘	↙
28 months	CXR	
32 months	CXR	
3 years	CXR, MRI or CT of the primary site, PE	
38 months	CXR	
42 months	CXR	
4 years	CXR, MRI or CT of the primary site, PE	
52 months	CXR	
56 months	CXR	
5 years	CXR, MRI or CT of the primary site, PE	

*CT* computed tomography, *CXR* chest X-ray, *MRI* magnetic resonance imaging, *PE* outpatient visit and clinical examination, *ULD-CT* ultra-low-dose computed tomography of the chest.

### Study protocol

This prospective study was approved by the Joint Ethics Committee of Helsinki University Hospital. The study was performed in accordance with the Declaration of Helsinki. After primary treatment with curative intent, patients fulfilling the inclusion criteria and giving written informed consent were enrolled between June 2017 and April 2019. Inclusion criteria included age > 18 years, primary non-metastatic high grade soft tissue sarcoma, and treatment with curative intent. In the study protocol, patients have their regular CXR once in two months plus ULD-CT imaging is repeated seven times during the first two years of follow-up with shortest interval in the beginning of follow-up (Table 1). ULD-CT and CXR were performed to the patients during the same visit to the radiology department. After two years, patients without detectable metastases continue in regular follow-up program.

#### Patients

Fifty patients with primary non-metastatic high grade soft tissue sarcoma treated with curative intention were included (Table 2). Undifferentiated pleomorphic sarcoma was the most common subtype. Six patients had a predisposing factor for sarcoma: two patients had an RB1 deletion, one patient had Li-Fraumeni syndrome, two patients with angiosarcoma had previous radiotherapy for breast cancer, and one patient with fibrosarcoma had a history of dermatofibrosarcoma protuberans. Two patients had a benign lesion in the primary staging pulmonary CT. Of these two benign lesions, one cleared, and one remained unchanged in ULD-CT during follow-up.

**Table 2:** Description of tumour, patient and treatment characteristics of 50 patients at presentation of the primary tumour.

Characteristics	No. of patients (%)
Sex	
Male	30 (60.0)
Female	20 (40.0)
Age at diagnosis (years)	
Median	63.6
Range	17.5–90.5
Primary tumour site	
Lower extremity	24 (48.0)
Upper extremity	11 (22.0)
Trunk	9 (18.0)
Retroperitoneum	3 (6.0)
Bronchus	1 (2.0)
Head and neck	1 (2.0)
Intra-abdominal	1 (2.0)
Histology	
UPS	13 (26.0)
NOS	8 (16.0)
Leiomyosarcoma	7 (14.0)
Liposarcoma	6 (12.0)
Myxofibrosarcoma	6 (12.0)
Synovial sarcoma	4 (8.0)
Angiosarcoma	3 (6.0)
Clear cell sarcoma	1 (2.0)
Epithelioid sarcoma	1 (2.0)
Fibrosarcoma	1 (2.0)
Depth	
Superficial	17 (34.0)
Deep	33 (66.0)
Tumor size (cm)*	
Median	5.0
Range	1.4–27
Margin	
Wide	27 (54.0)
Marginal	20 (40.0)
Intralesional	3 (6.0)
Radiation therapy	
Yes	23 (46.0)
No	27 (54.0)
Chemotherapy	
Yes	11 (22.0)
No	39 (78.0)

*NOS* not otherwise specified, *UPS* undifferentiated pleomorphic sarcoma.

*one missing.

#### CT scans

Altogether, 273 ultra-low-dose non-contrast chest CT scans were performed for 50 soft tissue sarcoma patients with a 64-slice CT scanner (GE Discovery CT750 HD, GE Healthcare, Milwaukee, WI, USA). The scan range was set from the apex of the lungs to the lateral phrenic angles. The scan protocol was modified from a low-dose chest CT phantom study^21^. The lowest dose protocol the radiation dose of which corresponds to a ULD-CT was used. Tube current ranges used for automated tube current modulation and tube voltages were 10–40 mA and 100 kVp and 10–50 mA and 120 kVp for patients weighting less and more than 60 kg, respectively. The GE noise index (NI), which is relative to the target image noise described as the standard deviation of CT numbers in Hounsfield Units (HU), was set to 80 in both protocols. Rotation time was 0.4 s, helical pitch 0.984, and detector configuration 8 × 5 mm. A model-based iterative reconstruction (VEO, GE Healthcare) algorithm was used to reconstruct 0.625 mm and 2.5 mm axial, and 3.0 mm coronal and sagittal images.

Volume CT dose index (CTDIvol) and dose-length product (DLP) of the scans were retrospectively collected from the dose reports. Effective doses of CT examinations were determined using a DLP to effective dose conversion coefficient of 0.0145 mSv/mGy cm^22^. The median and interquartile range (IQR, first quartile–third quartile) of the dose indices and effective dose were calculated.

## Results

**Figure 1:**
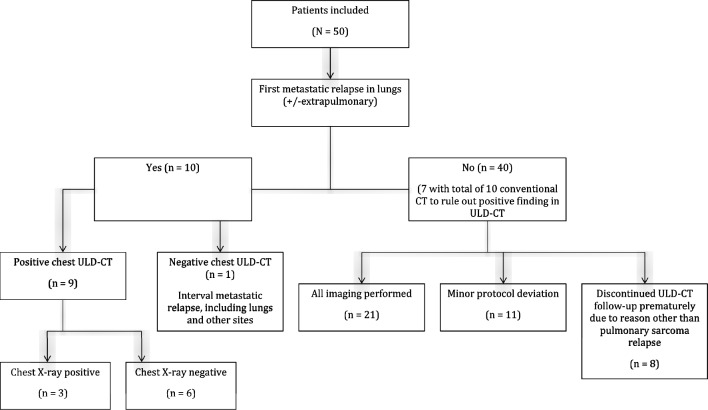
Flowchart of study population according to pulmonary relapse status and protocol compliance.

During follow-up, 13 (26%) patients had a relapse (Table 3). The median time to first distant relapse was six months. Of the 13 relapsing patients, eight patients had isolated pulmonary metastases, and two patients had both pulmonary metastases and metastases at other sites. Nine of these 10 pulmonary relapses were detected by ULD-CT and one due to symptoms between two controls (Fig. 1). Of the nine pulmonary relapses detected on ULD-CT only three were visible also at CXR performed on the same day.

**Table 3:** Details of the 13 relapsing patients.

Histology	Timing (mo)	Pattern of 1st relapse	Laterality	No of pulmonary nodules	Size of the largest pulmonary nodule (mm)	Detection mode	Treatment with curative intent	Status at FU (mo)
Sarcoma NOS	2	Pulmonary	Bi	4	9	ULD-CT	No	DOD (26 mo)
Sarcoma NOS	2	Pulmonary	Bi	12	12	ULD-CT+CXR	No	DOD (7 mo)
UPS	4	Pulmonary	Mono	4	5	ULD-CT	SBRT	NED (54 mo)
Myxofibrosarcoma	4	Pulmonary	Mono	1	9	ULD-CT+CXR	Surgery	DOD (12 mo)
Liposarcoma	6	Pulmonary	Mono	1	7	ULD-CT	Surgery	DOD (31 mo)
Sarcoma NOS	6	Pulmonary	Mono	1	14	ULD-CT+CXR	Surgery	DOD (30 mo)
Liposarcoma	6	Pulmonary and other sites and LR	Bi	> 10	5	ULD-CT	No	DOD (3 mo)
Leiomyosarcoma	10	Pulmonary	Bi	9	6	ULD-CT	No	DOD (48 mo)
Leiomyosarcoma	14	Pulmonary	Mono	1	6	ULD-CT	Surgery	DOD (30 mo)
Leiomyosarcoma	27	Pulmonary and other sites	Bi	61	5	symptoms	No	AWD (27 mo)
Angiosarcoma	10	Other sites*				symptoms	No	DOD (1 mo)
Myxofibrosarcoma	35	LR and lymph node				MRI	Surgery and XRT	DOD (21 mo)
Myxofibrosarcoma	12	LR				MRI	Surgery and XRT	NED (36 mo)

*AWD* alive with disease, *CXR* chest radiograph, *FU* follow-up, *DOD* dead of disease, *LR* local recurrence, nd no data, *MRI* magnetic resonance imaging, *NED* no evidence of disease, *NOS* not otherwise specified, *SBRT* stereotactic body radiotherapy, *ULD-CT* ultra-low-dose computed tomography, *UPS* undifferentiated pleomorphic sarcoma, *XRT* radiation therapy.

*liver and spleen.

The overall sensitivity for detection of pulmonary metastases by ULD-CT was 90% (9/10) and specificity 83% (33/40). Seven patients had uncertain findings on ULD-CT, which had to be verified with ten conventional CT investigations, or by further follow-up. The overall sensitivity of CXR was 30% (3/10).

### ULD-CT doses

Most studies were performed with 120 kVp tube voltage (225/273). The median CTDIvol (volume computed tomography dose index) was 0.52 mGy (IQR 0.38–0.83 mGy), with a range of 0.20 to 1.63 mGy. Similarly, the median DLP (dose-length product) was 18.96 mGy cm (IQR 13.48–29.42 mGy cm), range 8.12 to 61.10 mGy cm. The median effective dose from a single study was 0.27 mSv (0.20–0.43 mSv), range 0.12 to 0.89 mSv.

### Protocol discontinuations and deviations

In total 454 CXRs (range 1–12 per patient, mean 9, median 12) and 273 ULD-CTs (range 1–7 per patient, mean 5, median 7) were performed and assessed. Eight patients discontinued ULD-CT follow-up prematurely due to reason other than pulmonary sarcoma relapse: One patient discontinued because of deteriorated health, one developed symptomatic extrapulmonary relapse, one had a strong suspicion of metastatic relapse in lungs which was not later confirmed, one developed pulmonary sarcoidosis requiring follow-up with conventional chest CT, one patient moved abroad, two patients died unrelatedly, and one patient discontinued the study on her own request. These patients are included in the analyses until discontinuation. Twenty-one patients had a complete follow-up with no detected pulmonary metastasis. Eleven patients had minor deviations (one ULD-CT not performed, n = 8) from the follow-up protocol, all these patients were included in the analysis. Of the 40 patients with no pulmonary relapse, seven patients had a total of ten conventional pulmonary CTs to evaluate suspicious lesions detected in ULD-CT.

### Treatment and outcome of relapsing patients

**Figure 2:**
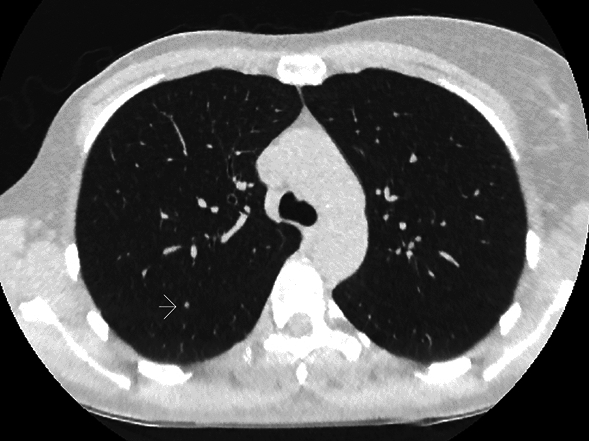
Ultra-low-dose CT (ULD-CT) image of a patient for whom radiologist considered the ULD-CT finding diagnostic and verification with normal dose contrast enhanced CT unnecessary. Slice shows one of the four metastatic nodules of all which were invisible in chest X-ray.

**Figure 3:**
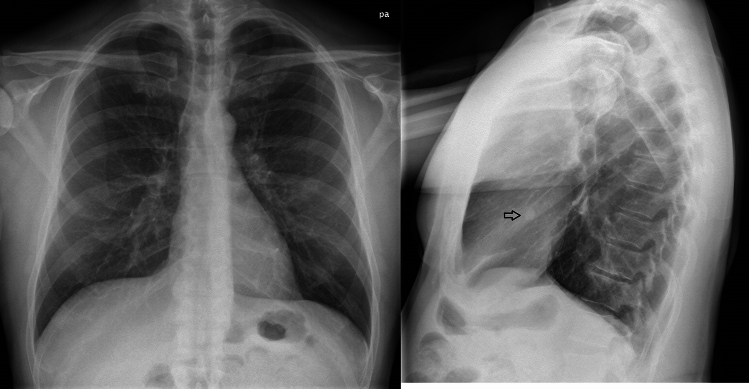
Chest X-ray (CXR) of a patient with 12 metastatic nodules visible in ULD-CT at 2 months. CXR shows the largest nodules (12 mm).

Nine patients had an asymptomatic pulmonary relapse detected in ULD-CT. Seven were confirmed with normal dose contrast enhanced CT. For two patients, radiologist considered the ULD-CT finding diagnostic, and verification with normal dose contrast enhanced CT unnecessary (Fig. 2). These two patients had a bilateral pulmonary relapse at 2 months with 4 and 12 nodules measuring up to 9 mm and 12 mm, respectively (Fig. 3). Both patients died of the disease (at 9 and at 26 months, respectively).

Five of the nine patients were considered unresectable, due to large extent of pulmonary metastases or extrapulmonary disease. Four patients were operated on, but all developed new metastases, and later died due to disease. One patient with four nodules up to 5 mm had an SBRT course of 5 × 5 Gy and remained disease free at 54 months after therapy.

Both patients diagnosed with an isolated locoregional recurrence were treated surgically with curative intention. The third patient diagnosed with a locoregional recurrence and simultaneous unresectable metastases at multiple sites had palliative chemotherapy.

One patient diagnosed with interval symptomatic metastatic pulmonary relapse with metastases also in several locations outside the lungs received palliative chemotherapy and remains alive with disease. One patient with interval symptomatic metastatic extrapulmonary relapse had best supportive care only due to poor general health.

## Discussion

ULD-CT was more sensitive than CXR in detecting pulmonary metastases in this prospective trial in patients with high grade soft tissue sarcoma. The median time to first distant relapse was only six months. This is to our knowledge the first report of follow-up with ULD-CT in sarcoma patients. The diagnostic accuracy of ULD-CT in lung lesions has been shown to be comparable to conventional CT^23^. A few studies have, however, compared conventional pulmonary CT to CXR in the follow-up of soft tissue sarcoma patients^6,7,24^.

A prospective randomized Indian study comprising both bone (n = 359) and soft tissue sarcomas (n = 141) demonstrated the higher sensitivity of CT in detecting pulmonary relapse^6,7^. Median time to first pulmonary metastasis was approximatively 8 months in the CT group compared to 14 months in the CXR group (times estimated from graph). However, in this study, earlier detection did not translate into improved survival. In a retrospective series from Seoul, Korea, a tendency of earlier detection of pulmonary metastases was recorded in CT group versus CXR group (14.5 months versus 17 months) during follow-up, but 5-year survival in these groups did not differ^24^. Altogether these studies indicate that pulmonary CT is more sensitive in detecting pulmonary relapse in sarcoma patients than CXR. The present study points to that this is the case also for ULD-CT as only three of the nine asymptomatic pulmonary relapses were visible also at CXR taken on the same day.

Whether the earlier detection of relapse in patients with soft tissue sarcoma would lead to improved (long-term) patient outcome is uncertain. A proportion of sarcoma patients relapsing either locally or in the lungs may also be offered potentially curative surgical treatment. This is the main rational for follow-up of sarcoma patients with regular imaging of the primary site and the lungs because the lungs are the most frequent site of first distant relapse in sarcomas^25–31^.

In a previous study from our department comprising 347 relapses in 1,580 soft tissue sarcoma patients, 41% had their first relapse in the lungs, and of these 51% had surgery^3^. The proportion of cases with first relapse including the lungs was higher in the present study, which may partly be due to the higher sensitivity of ULD-CT compared to imaging used in this older series. In the present study, five out of ten patients were eligible for local treatment (surgery or SBRT), which is comparable to the proportion in our previously published retrospective series^3^.

The retrospective series from Seoul strongly suggested that earlier detection of lung metastases with CT would facilitate resectability^24^. Three other studies of sarcoma follow-up with conventional imaging also indicated that early detection of pulmonary metastases may facilitate resections and improve outcome^26,30,32^. In the first two studies reporting altogether 85 isolated pulmonary relapses, 33 out of 58 asymptomatic patients were eligible for surgery whereas none of the 27 patients with relapse detected by symptoms were eligible^26,30^. Patients undergoing surgery had markedly longer median survival. In a retrospective series from Taipei, Taiwan, more frequent follow-up was associated with improved survival in high-risk patients diagnosed with a systemic relapse by enabling metastasectomy^32^. On the other hand, the prospective study by Puri et al. failed to find any impact on outcome by early detection. Data on the treatment of relapses, especially the frequency of therapeutic interventions with a curative intention, was not disclosed^6,7^. Thus, it remains uncertain if earlier detection by ULD-CT would improve resectability of pulmonary metastases or overall outcome.

Earlier detection gives more time for treatment planning, including staging investigations, assessment of vital functions, and suitability for surgery before the disease progresses beyond operable stage. This could be achieved without exposing the patient to significantly higher doses of ionizing radiation, the total amount of irradiation of the 7 investigations in the present trial is 1.89 mSv, equivalent to 38 CXR. The median effective dose from a single ultra-low-dose chest CT examination was determined to be 0.27 mSv (0.20–0.43 mSv). This is only five times the dose level of a conventional chest X-ray at our institution, 0.05 mSV, which is somewhat lower than reported previously (from 0.02 mSv [posterior-anterior projection study] to 0.1 mSv [a study containing both posterior-anterior and lateral projections])^33^. The effective dose was comparable to dose levels (0.16–0.38 mSv) achieved in a low-dose chest CT phantom study^21^. However, Kaasalainen et al.^21^ used higher DLP to effective dose conversion coefficient (k = 0.024 mSv/mGy·cm) than in this study (k = 0.0145 mSv/mGy·cm). This was due to geometrical and gender differences between the study subjects.

The main strengths of the present study are its prospective design and relatively high degree of compliance. This study has also several limitations. Firstly, only one CT scanner from a single vendor was used. As image reconstruction algorithms and other optimization tools differ between the CT systems, the results may not be generalizable to other CT devices. Another weakness is the small number of patients and, therefore, the small number of relapses. Moreover, 14% of patients discontinued ULD-CT prematurely, most due to health problems, which is not surprising in this old patient population. This had, however, little impact on the results since none of the patients who discontinued experienced a pulmonary relapse. Furthermore, since both imaging methods were done on the same day any impact on imaging results on treatment intervention and outcome was not possible to analyze. ULD-CT was restricted to the first 2 years of the planned 5-year follow-up period. As 10 of the 12 distant relapses and all pulmonary relapses occurred within this period, this 2-year period of intensified follow-up seems appropriate. A main drawback of ULD-CT, besides the higher radiation dose is the higher costs of imaging. The cumulative costs of the seven ULD-CTs in the trial protocol were 1008 € per patient, equivalent to the cost of 38 CXRs. Replacing pulmonary imaging in follow-up of soft tissue sarcoma patients on a larger scale with ULD-CT would require a clear additional investment from the service system, both in equipment investments and in the form of image interpretation. Based on presently reported data we have not altered our follow-up protocol. The present study was not powered to detect any survival benefit, but it seems unlikely that in the study protocol ULD-CT would offer a plausible option to improve the survival of the patients with the most aggressive systemic disease albeit earlier detection of pulmonary relapse. Many reports have reported better survival in patients with pulmonary relapse after an interval longer than 12 months after primary treatment probably pointing to less aggressive tumour biology^34^.

Our study demonstrated that ultra-low-dose CT outperforms CXR in the detection of pulmonary metastases of sarcoma. ULD-CT combines the increased accuracy of CT with a low radiation dose. Further research is needed to accurately determine how ULD-CT could improve the survival of sarcoma patients. Moreover, efforts should be made to identify subgroups of sarcoma patients at increased risk of pulmonary metastases, who would most likely benefit from ULD-CT-based follow-up.

### Supplementary Information

Supplementary Information.

## Data Availability

The datasets used and/or analyzed during the current study are available from the corresponding author on reasonable request.
