# Supplementary material for: Ultra-low-dose computed tomography and chest X-ray in follow-up of high-grade soft tissue sarcoma—a prospective comparative study
Source: Sci Rep. 2024 Mar 26;14:7181. doi: 10.1038/s41598-024-57770-z (PMC10965957; doi:10.1038/s41598-024-57770-z)
Supplement: Supplementary file 1 — Supplementary Information. [file 41598_2024_57770_MOESM1_ESM.docx]

Ultra-low-dose computed tomography and chest x-ray in follow-up of high-grade soft tissue sarcoma - a prospective comparative study

Samuli Salminen, Sari Jäämaa, Riikka Nevala, Markus J. Sormaala, Mika Koivikko, Erkki Tukiainen, Jussi Repo, Carl Blomqvist, Mika Sampo


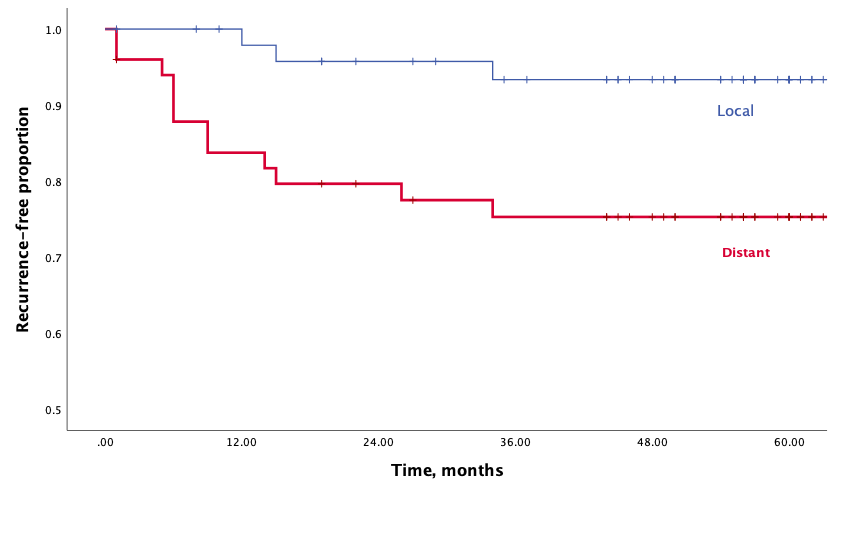


Supplementary Figure 1. Time to local or distant recurrence (Kaplan-Meier analysis).
